# Improving Cognitive Function after Traumatic Brain Injury: A Clinical Trial on the Potential Use of the Semi-Immersive Virtual Reality

**DOI:** 10.1155/2019/9268179

**Published:** 2019-07-30

**Authors:** Rosaria De Luca, Maria Grazia Maggio, Giuseppa Maresca, Desiree Latella, Antonino Cannavò, Francesca Sciarrone, Emanuele Lo Voi, Maria Accorinti, Placido Bramanti, Rocco Salvatore Calabrò

**Affiliations:** IRCCS Centro Neurolesi “Bonino Pulejo”, Messina, Italy

## Abstract

Traumatic brain injury (TBI) is the most common cause of long-term disability and death among young adults, and it represents an enormous socioeconomic and healthcare burden. Our purpose is to evaluate the effects of a virtual reality training with BTs-Nirvana (BTs-N) on the recovery of cognitive functions in TBI subjects, using the interactive semi-immersive program. One hundred patients with TBI were enrolled in this study and randomized into either the Traditional Cognitive Rehabilitation Group (TCRG: *n* = 50) or the Virtual Reality Training Group (VRTG: *n* = 50). The VRTG underwent a VRT with BTs-N, whereas the TCRG received a standard cognitive treatment. Each treatment session lasted 60 minutes and was repeated three times a week for 8 weeks. All of the patients were evaluated by a specific psychometric battery before (T0) and immediately (T1) after the end of the training. VRTG and TCRG had a significant improvement in cognitive functioning and in mood, but only VRTG presented with a significant increase in cognitive flexibility and shifting skills and in selective attention. In conclusion, our results suggest that VR may be a useful and effective approach for the rehabilitation of patients with TBI, leading to better cognitive and behavioral outcomes.

## 1. Introduction

Traumatic brain injury (TBI) is a condition caused by a mechanical event that causes skull and/or brain damage due to a strong and violent head blow (i.e., falls and sport injuries), strong rotations of the head (i.e., road accidents), or penetration of objects in the cranium (i.e., bullets) thus causing focal or diffuse damage to multiple brain areas [1]. TBI is the most common cause of long-term disability and death among young adults, and it represents an enormous socioeconomic and healthcare burden [2]. It is estimated that about 5.48 million people suffer from severe TBI each year (73 cases per 100,000 people) [3]. Among the survivors of moderate to severe head injury, 31.8% of patients die or need hospitalization in a specialized health center; 44% are unable to return to work, and 88% of the patients with mild TBI have white matter damage, with negative repercussions on functional outcomes [4]. In fact, TBI may affect motor, cognitive, emotional, and psychological functions with a consequent worsening of both patient and his/her caregiver's quality of life [5]. In particular, cognitive dysfunction may interfere with work, relationships, leisure, and daily activities, increasing the burden of the disease [6, 7]. Growing evidence demonstrates that cognitive rehabilitation (CR), through previously learned skills or new compensatory strategies, is effective in patients with TBI as it enhances cognitive and psychosocial interaction [8–12]. In recent years, technological innovations have allowed the development of new rehabilitative strategies, such as PC-based rehabilitation or Virtual Reality Training (VRT), which have proven effective in the CR of neurological patients [13–16]. Chen et al. [17], examining the efficacy of PC-based rehabilitation in TBI subjects, observed significant posttreatment improvements on cognitive domains. Moreover, it has been demonstrated that PC cognitive training can be a potential CR strategy to optimize cognitive and global functional recovery [18, 19]. Several studies using VR have shown that it increases cognitive and behavioral skills in patients with TBI [13–16]. Indeed, it has been demonstrated that VR may be effective in improving executive functions in patients with TBI in the subacute phase [20]. In a recent review, Maggio et al. [9] found that VR might positively affect memory, attention, executive function, behavior, and mood in individuals with TBI. Indeed, evidence of the use of VR in TBI cognitive neurorehabilitation is very poor and there is not enough consensus on its use in the context of TBI rehabilitation [13].

The aim of this study is to evaluate the effects of a VRT using BTs-Nirvana (BTs-N) for the recovery of cognitive and behavioral functions in patients with TBI through an interactive semi-immersive program.

## 2. Material and Methods

### 2.1. Study Population

One hundred patients with TBI (mean ± SD age: 39.93 ± 10.1 years; 56% males), who attended our Behavioral and Robotic Neurorehabilitation Service from January 2016 to December 2018, were enrolled in this study and randomized in order to be recruited into either the Traditional CR Group (TCRG: *n* = 50) or the VRT Group (VRTG: *n* = 50) (Table 1). Inclusion criteria were (i) neurological diagnosis of mild to moderate TBI in the postacute phase (i.e., 3 to 6 months from the acute event), (ii) ability to sit for at least 20 minutes (including at least one minute without support), and (iii) presence of mild to moderate cognitive impairment (Montreal Cognitive Assessment (MoCA) from 18 to 25 [21]). Exclusion criteria were (i) age > 85 years, (ii) presence of disabling sensory alterations and frequent episodes of recurrent epilepsy (especially positive symptoms such as audio-video hallucination), and (iii) concomitant medical and psychiatric illness possibly interfering with the VR training.

### 2.2. Study Design

**Figure 1:**
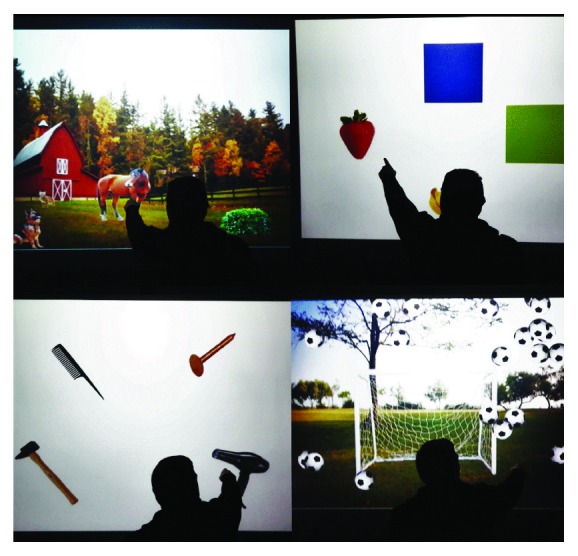
A patient affected by traumatic brain injury performing cognitive training in the semi-immersive virtual scenario created by BTs-Nirvana.

All of the patients underwent the same amount of CR, but using different tools. TCRG underwent traditional CR, administered in individual sessions using a face-to-face interaction between therapist and patient with paper and pencil activities, whereas VRTG performed a VRT using BTs-N. VR allows a multisensory and interactive simulation of scenarios that affect real life with the aid of a computer. The recreated situations are generally three-dimensional and reproduce real objects and events, improving the cognitive abilities of patients. In particular, BTs-N is a semi-immersive therapy program, for motor and cognitive rehabilitation, which offers interactive virtual scenarios in which patient carries out the training with the help of a therapist. The patient interacts with virtual scenarios and audio-visual stimuli through movement, creating a total sensory involvement that facilitates rehabilitation of attention, visual-spatial, and executive skills (Figure 1). All patients (TCRG and VRTG) underwent a total of 24 1 h sessions (3 times a week for 8 weeks). Both groups underwent the same conventional physiotherapy program, aimed at improving muscle strength, coordination, and spasticity. The detailed rehabilitative program in both groups is described in detail in Table 2.

### 2.3. Outcome Measures

Each participant was assessed by means of a neuropsychological evaluation before (T0) and immediately after the end of the training (T1). A skilled neuropsychologist administered a battery of tests including Montreal Cognitive Assessment (MoCA) [21] to assess the general cognitive state; Hamilton Rating Scale Depression (HRS-D) [22] and Hamilton Rating Scale Anxiety (HRS-A) [23] to assess mood and anxiety, respectively; Frontal Assessment Battery (FAB) [24] and Weigl's Test [25] to evaluate frontal abilities; and Visual Search (VS) [26] and Trial Making Test (TMT) [27] to measure the attention process, attentive shifting, and visual research abilities.

The present study was conducted in accordance with the 1964 Helsinki Declaration and approved by our Research Institute Ethics Committee (ID 25/2015); written informed consent was obtained from all participants.

### 2.4. Data Analysis

Data were analyzed using the SPSS 16.0 version, considering a *p* < 0.05 as statistically significant. Using SPSS, we performed the analysis of variance (ANOVA) in order to assess whether the type of treatment influenced the clinical outcome, independently from the score difference at baseline. The dependent variable consisted in the performances obtained in tests of the different cognitive functions; the categorical variable was the “Group” (1 = VRTG; 2 = TCRG); instead, the variable was “Time” (factor within the subject with two levels: T0 and T1). Finally, the independent variable consisted of scales/tests to evaluate neuropsychological functions and mood. Student's *t*-tests, using the Bonferroni correction, were used for post hoc testing of group differences in time and performance.

## 3. Results

All of the patients completed the training program without any adverse events, including cyber-sickness. No significant differences were found in age (*p* = 0.22), sex (*p* = 0.22), and education (*p* = 0.69) between VRTG and TCRG. At baseline, no significant differences emerged between the test scores of the two groups. The ANOVA showed the triple interaction between Group^∗^Time^∗^Tests/Scales (*F*(_9162_) = 21741, *p* < 0.001). In particular, ANOVA decomposition (Table 3) highlighted how the effect of the two treatments was significantly different, influencing the scores of all tests/scales. Post hoc analysis results (Table 4) showed that VRTG and TCRG had a significant improvement in various cognitive functioning and mood. However, we observed a significant increase in cognitive flexibility and shifting skills (TMT B-A) and in selective attention/visual research (VS) only in the VRTG. Moreover, at T1, we found a significant difference between VRTG and TCRG for all of the test scores, with a greater improvement in VRTG, except for anxiety (HRS-A), whose improvement was similar in both groups.

## 4. Discussion

Our data confirm that VRT may be effective in the recovery of patients with TBI. In fact, although both groups achieved significant improvements in different cognitive and mood domains, patients undergoing VRT obtained better results. Moreover, only VRTG improved in specific cognitive domains, such as cognitive flexibility, attentional shifting, visual search, and executive and visuospatial functions, that are necessary for planning and managing daily life. Thus, VR can be considered a useful tool for patients with TBI, as demonstrated in various neurological disorders by previous studies [28–33]. Indeed, it has been shown that VRT is effective in enhancing attention, visual-spatial capacity, and motor function in patients with stroke [28, 29]. Doniger et al. showed that VR is a useful tool in cognitive and motor rehabilitation of patients with Alzheimer's disease [30]. These positive results were also confirmed in patients with multiple sclerosis, as 2D VR was able to further boost neural plasticity and thus functional recovery [31]. A recent pilot study performed on individuals with Parkinson's disease found that VR improved cognitive functions, with regard to executive and visuospatial domains, besides mood [32]. Finally, Maresca et al. observed significant improvements in different cognitive and motor domains as well as a reduction in anxiety and depressive symptoms in a patient affected by spinal cord injury [33]. Our data confirm these results, demonstrating that VRT can be effective also for patients with TBI. In our study, we used BTs-N, which creates a three-dimensional computer environment that can be explored using computer devices, projecting the user into a realistic scenario. This experience promotes the whole involvement of the patient, as the increased feedback may induce major changes in neuronal plasticity that are responsible for restoring motor activity and/or cognitive function, thanks to the so-called “reinforcement learning” [28]. This leads us to believe that VRTG registered better results because VR may have boosted the neural plasticity processes and thus the functional recovery, as compared to the traditional therapies. In fact, it is well known that physical and cognitive exercise can increase the process of brain repair and plasticity after injuries, and the recovery is better, more intensive, repetitive, and task-oriented [34, 35]. The brain areas most often involved in TBI are the frontal and temporal lobes, especially in the basal areas and the subcortical white matter, leading to attention deficit, learning and memory, affect and expression, problem solving abilities, and executive function with a significant impact on the quality of life of the patient and his/her family [5, 6]. Therefore, VRT, thanks to the multisensory approach, can stimulate and enhance the spontaneous post-TBI regeneration processes, which otherwise may be short-lived and too weak to counter the deterioration of damage [36]. In fact, the exercises performed in a virtual environment help the patient to develop the knowledge of the results of the movements (knowledge of the results) and the knowledge of the quality of the movements (knowledge of the performances), which positively affect patient's functional recovery, including the cognitive one [28]. Thus, VR could allow greater results than paper-pencil exercises, through global stimulation and dual cognitive and motor tasking, which allow greater patient involvement. Indeed, according to Dahdah et al. [20], our data demonstrate, for the first time ever, that semi-immersive VR may be effective in improving executive functions and the speed of information processing in patients with TBI. Furthermore, VR increases motivation and enjoyment of the patients (important factors for successful rehabilitation), further favoring the behavioral and cognitive recovery, as also observed by Dvorkin et al. [37].

The main limitation of the study is the absence of a control group without a cognitive treatment, to exclude the case that patients' mood, anxiety, and some aspects of cognition improved due to recovery independent of the interventions. Nonetheless, this study design is difficult to perform, as CR is becoming the standard treatment of neurological patients.

## 5. Conclusions

This study suggests that semi-immersive VR using BTs-N may be a useful approach for the rehabilitation of individuals with TBI, potentially leading to better cognitive and behavioral outcomes. Further studies are needed, to confirm our promising results and to assess whether and to what extent VR cognitive training can improve overall functional recovery and quality of life in TBI patients.

## Figures and Tables

**Table 1:** Demographic characteristics at baseline for both of the groups.

	Virtual Reality Training Group	Traditional Cognitive Rehabilitation Group	All	*p* value
Participants	50	50	100	
Age	38.7 ± 9.3	41.1 ± 10.8	39.9 ± 10.1	0.22
Education	2.9 ± 0.8	2.7 ± 0.8	2.8 ± 0.850	0.23
Gender				0.69
Male	29 (57.9%)	26 (52%)	56 (56%)	
Female	21 (42.1%)	24 (48%)	44 (44%)	
Interval from TBI				
Mean in months	4.5 ± 1.5	4 ± 2	4.7 ± 1.3	0.78
Brain lesion site/side				
Cortical right	22	24	46	
Subcortical right	16	17	33	
Cortical left	8	6	14	
Subcortical left	4	3	7	

Quantitative variables were expressed as means ± standard deviations, categorical variables as frequencies and percentages.

**Table 2:** TBI cognitive rehabilitative program.

Cognitive domain	Conventional cognitive rehabilitation	Cognitive training by BTs-Nirvana
Executive functions	The patient uses tools, such as a pencil, sheets, and a pen, to perform exercises in a specific physical space (rehabilitation table); the exercises can also provide tasks of simple associations (i.e., letter-color), inhibitory control, and arithmetic operations; estimating the numerical quantity and the categorization; and the deductive logical reasoning.	The patient performs exercises in a virtual environment through the movement performed in the interactive screen. The movements allow to move or manipulate specific objects, in different directions (i.e., balls, flowers, and butterfly), or to create specific associations (i.e., color-number) with a dynamic interaction in the virtual environment. When the patient touches the virtual objects, he/she determines an audio and video feedback (using the sprite activity). In particular, the subject can perform ideomotor sequences with the guidance of the therapist, calculation and numerical processing, inhibitory control, and arithmetic operations; can estimate the numerical quantity and the categorization; and can perform the deductive logical reasoning, using a specific virtual task.
	*Rehabilitative resources*: face-to-face rehabilitative session between the patient and the therapist, using paper and pencil materials.	*Virtual scenarios*: The Bricklaying Tools; Hopscotch; The Colour of Fruit; Walk Through; and Eggs Circle.
	Each training is divided into three different levels of difficulty in relation to the complexity of the tasks, the number of errors, or the speed of execution of the exercise.	The difficulty level increases (from the first to the third level) with the increment of the complexity of the virtual task, elements on the screen, and greater difficulty of the requests by the therapist.
Attention processes and visual-spatial cognition	The patient must indicate and touch specific target stimuli in relation to specific characteristics (color, image, animal, function, etc.), neglecting the distractors. The therapist gives verbal deliveries to the patient, combining the image corresponding to the selection (which reflects the characteristics of the objects to choose).	The patient selects/explores some elements (colors, musical arcs, geometric shapes or not, animals, etc.) observed in the virtual environment. These elements remain visible to the observer for a variable time, established by the interaction between the virtual system, the therapist, and the patient. The patient touches the virtual target element, at a specific time; this action causes a visual change with a typical audio/video feedback (positive reinforcement); otherwise, the element disappears (negative reinforcement) (Hunt task).
	*Rehabilitative resources*: face-to-face rehabilitative session between patient and therapist, use of paper and pencil materials.	*Virtual scenarios*: Billiards, Piano, Storm, Flowerized, and Goal.
	*Cognitive therapist structures tasks with increased difficulty, according to specific parameters such as number and type target, number and type of distractor, and complexity of consign.*	*The level of difficulty increases with the increase of the numbers of distractors and reducing the usable time of execution.*

**Table 3:** ANOVA decomposition in Group^∗^Time for all tests/scales.

	Degree of freedom	Mean square	*F*	*p* value
MoCA	1, 98	100.82	242.76	**<0.001**
HRS-D	1, 98	129.60	54.45	**<0.001**
HRS-A	1, 98	88.44	40.76	**<0.001**
TMT-A	1, 98	883.42	17.35	**<0.001**
TMT-B	1, 98	20555.60	56.78	**<0.001**
TMT B-A	1, 98	7082.28	21.21	**<0.001**
VS	1, 98	571.119	43.72	**<0.001**
FAB	1, 98	52.92	60.61	**<0.001**
WEIGL	1, 98	86.39	69.28	**<0.001**

Significant *p* values are in bold. Legend: FAB: Frontal Assessment Battery; HRS-A: Hamilton Rating Scale for Anxiety; HRS-D: Hamilton Rating Scale for Depression; MoCA: Montreal Cognitive Assessment; TMT-A: Trail Making Test—Form A; TMT-B: Trail Making Test—Form B; TMT B-A: Trail Making Test—Form B-A; VS: Visual Search; WEIGL: Weigl Test.

**Table 4:** Post hoc analysis of clinical scores between baseline (T0) and follow-up (T1), for both the Virtual Reality Training Group (VRTG) and the Traditional Cognitive Rehabilitation Group (TCRG).

Clinical assessment	VRTG		*p* value	TCRG		*p* value
	T0	T1		T0	T1	
MoCA	23.0 (21.25–24.7)	27.0 (26.0–28.0)	**<0.001**	23.0 (20.0-24.7)	24.0 (22.0-25.7)	**<0.001**
HRS-D	10.0 (6–13.7)	5.0 (3.0–7.0)	**<0.001**	12.0 (7.25-13.0)	10.0 (6.25-12.0)	**<0.001**
HRS-A	10.0 (4.25–13.7)	6.0 (1.0–8.7)	**<0.001**	9.0 (5.25-11.7)	7.0 (5.0-10.0)	**<0.001**
TMT-A	67.5 (55.25-100)	57.0 (35.0–88.0)	**<0.001**	79.5 (57.25-168.0)	74.5 (55.0-160.75)	**<0.001**
TMT-B	201.5 (130.2–274.0)	145.5 (92.0–200.0)	**<0.001**	179.0 (140.0-246.5)	174.0 (140.0-237.5)	**<0.001**
TMT B-A	95.5 (57.5–161.0)	82.5 (40.0–115.5)	**<0.001**	82.0 (65.0-160.5)	80.5 (62.5-155.0)	0.4
VS	34.0 (26.0–43.7)	42.7 (36.8–47.2)	**<0.001**	33.6 (25.1-43.7)	36.8 (27.1-46.2)	**0.002**
FAB	14.4 (11.1–15.9)	17.2 (15.2–18.0)	**<0.001**	13.6 (13.0-16.3)	14.9 (14.0-16.4)	**<0.001**
WEIGL	8.1 (6.3–9.2)	12.1 (10.1–14.0)	**<0.001**	7.2 (4.7-10.7)	8.2 (5.8-11.5)	**<0.001**

Scores are in median (first-third quartile); significant differences are in bold. Legend: FAB: Frontal Assessment Battery; HRS-A: Hamilton Rating Scale for Anxiety; HRS-D: Hamilton Rating Scale for Depression; MoCA: Montreal Cognitive Assessment; TMT-A: Trail Making Test—Form A; TMT-B: Trail Making Test—Form B; TMT B-A: Trail Making Test—Form B-A; VS: Visual Search; WEIGL: Weigl Test.

## Data Availability

The data used to support the findings of this study are available from the corresponding author upon request.
